# Innate Lymphoid Cells in the Maternal and Fetal Compartments

**DOI:** 10.3389/fimmu.2018.02396

**Published:** 2018-10-26

**Authors:** Derek Miller, Kenichiro Motomura, Valeria Garcia-Flores, Roberto Romero, Nardhy Gomez-Lopez

**Affiliations:** ^1^Perinatology Research Branch, Division of Obstetrics and Maternal-Fetal Medicine, Division of Intramural Research, Eunice Kennedy Shriver National Institute of Child Health and Human Development, National Institutes of Health, U. S. Department of Health and Human Services, Bethesda, MD and Detroit, MI, United States; ^2^Department of Obstetrics and Gynecology, Wayne State University School of Medicine, Detroit, MI, United States; ^3^Department of Obstetrics and Gynecology, University of Michigan, Ann Arbor, MI, United States; ^4^Department of Epidemiology and Biostatistics, Michigan State University, East Lansing, MI, United States; ^5^Center for Molecular Medicine and Genetics, Wayne State University, Detroit, MI, United States; ^6^Department of Immunology, Microbiology and Biochemistry, Wayne State University School of Medicine, Detroit, MI, United States

**Keywords:** amniotic cavity, decidua, LTi, maternal-fetal interface, neonate, pregnancy, preterm labor, uterus

## Abstract

Pregnancy success is orchestrated by the complex balance between the maternal and fetal immune systems. Herein, we summarize the potential role of innate lymphoid cells (ILCs) in the maternal and fetal compartments. We reviewed published literature describing different ILC subsets [ILC1s, ILC2s, ILC3s, and lymphoid tissue inducer (LTi) cells] in the uterus, decidua, fetal tissues [liver, secondary lymphoid organs (SLO), intestine, and lung] and amniotic cavity. ILC1s, ILC2s, and ILC3s are present in the murine uterus prior to and during pregnancy but have only been detected in the non-pregnant endometrium in humans. Specifically, ILC2s reside in the murine uterus from mid-pregnancy to term, ILC1s increase throughout gestation, and ILC3s remain constant. Yet, LTi cells have only been detected in the non-pregnant murine uterus. In the human decidua, ILC1s, ILC3s, and LTi-like cells are more abundant during early gestation, whereas ILC2s increase at the end of pregnancy. Decidual ILC1s were also detected during mid-gestation in mice. Interestingly, functional decidual ILC2s and ILC3s increased in women who underwent spontaneous preterm labor, indicating the involvement of such cells in this pregnancy complication. Fetal ILCs exist in the liver, SLO, intestine, lung, and amniotic cavity. The fetal liver is thought to be the source of ILC progenitors since the differentiation of these cells from hematopoietic stem cells occurs at this site, and mature ILC subsets can be found in this compartment as well. The interaction between LTi cells and specialized stromal cells is important during the formation of SLO. Mature ILCs are found at the mucosal surfaces of the lung and intestine, from where they can extravasate into the amniotic cavity. Amniotic fluid ILCs express high levels of RORγt, CD161, and CD103, hallmarks of ILC3s. Such cells are more abundant in the second trimester than later in gestation. Although amniotic fluid ILC3s produce IL-17A and TNFα, indicating their functionality, their numbers in patients with intra-amniotic infection/inflammation remain unchanged compared to those without this pregnancy complication. Collectively, these findings suggest that maternal (uterine and decidual) ILCs play central roles in both the initiation and maintenance of pregnancy, and fetal ILCs participate in the development of immunity.

## Introduction

Successful pregnancy requires the participation of numerous immune cell subsets that must be maintained at perfect equilibrium in the maternal and fetal compartments (1, 2). Both innate and adaptive immune cells have been shown to play important roles in the maintenance and completion of pregnancy (3, 4). The discovery of innate lymphoid cells (ILCs), which bridge the innate and adaptive immune systems, has opened up a new field of investigation with the potential to further uncover the complex immune state of pregnancy.

Innate lymphoid cells (ILCs) are defined by the following characteristics: (1) a lack of antigen-specific receptors, (2) the absence of the expression of known immune cell lineage markers, and (3) lymphoid cellular morphology (5–7). ILCs were divided into three primary groups based on their phenotype and functions (6). Type 1 ILCs (ILC1s) include the prototypical natural killer (NK) cells as well as non-cytotoxic IFNγ-producing ILC1s, identified by expression of the transcription factor T-bet (6, 8, 9). Type 2 ILCs (ILC2s) function through the release of type 2 cytokines such as IL-5 and IL-13 (10–15) and are thought to rely primarily on GATA-binding protein 3 (GATA3) and retinoic acid receptor-related orphan receptor-α (RORα) during their differentiation (6, 16, 17). These ILC2s participate in immune responses such as parasitic infection (12) and allergy (18) but also serve as systemic regulators of homeostasis (19–21). Type 3 ILCs (ILC3s) were divided into two main groups: lymphoid tissue inducer (LTi) cells and non-LTi ILC3s, referred to hereafter as ILC3s (6). LTi cells are critical for the formation of secondary lymphoid organs (SLO) and isolated lymphoid tissues (i.e., Peyer's patches) during fetal development (22–26). Such cells are also found in adults, where they are referred to as LTi-like cells since they do not generate new lymphoid tissue (27). LTi cells and ILC3s rely on expression of RORγt for their development (26) and can express IL-17A and/or IL-22; however, multiple ILC3 subsets with slightly different phenotypes and functional profiles have been described (6). Moreover, a degree of plasticity exists between ILCs, creating an additional layer of complexity within the ILC family (28–31). Recently, it was proposed that the classification of ILCs be expanded to five subsets in order to reflect their distinct developmental pathways: NK cells, ILC1s, ILC2s, ILC3s, and LTi cells (7).

In this review, we aimed to highlight the potential roles of ILCs in the uterus, the decidua, which is the site of direct contact between the maternal and fetal (chorion or trophoblast) tissues, the fetal organs, and the amniotic cavity. Within the field of perinatal immunology, it has been established that uterine (decidual) NK cells play important roles in the maintenance of pregnancy, and their functions are well reviewed elsewhere (32–35). Recent studies have shown that the other ILC subsets exist in the maternal and fetal tissues, suggesting that they also contribute to pregnancy maintenance and outcome. Therefore, in this review, we have focused on ILC1s, ILC2s, ILC3s, and LTi cells. Despite recent advances in the study of ILCs during pregnancy, several gaps still exist in the current knowledge. This review may provide insight into the known roles of ILCs during pregnancy and reveal new potential areas for future studies.

## Uterine innate lymphoid cells

Over the last decade, valuable information has been provided about the presence of ILCs in both the non-pregnant and pregnant uterus. A subset of ILC-like cells was first described in the human uterine mucosa (36). Such cells were originally considered precursors to uterine NK cells, yet showed a divergent phenotype and functionality through the expression of ILC3- and LTi-specific genes such as *RORC, LTA*, and *IL22* (36), indicating a different role for these cells. These results were confirmed later by the detection of ILC1s (37), ILC2s (38), and ILC3s (37, 38) in the human non-pregnant endometrium and reinforced by the demonstration that such cells are present in the murine uterus during pregnancy as well (37–41). Such studies have formed a foundation for the understanding of uterine ILCs; yet, future research is needed to further elucidate the role of these cells during pregnancy.

## Uterine ILC1s

Uterine ILC1s were first described in non-pregnant mice as a distinct subset of NK-like cells (42). This ILC1-like population was maintained in the murine uterus of *Nfil3*^−/−^ and *Tbet*^−/−^ mice, whereas NK cell subsets were affected (42). The transcription factors Nfil3/E4BP4 (43–45) and Tbet (6, 8, 9, 46) are both thought to be important for general NK and ILC development; thus, this study indicates that uterine ILC1s may have alternative developmental pathways. This study was confirmed by the detection of a similar ILC1 subset in the uterine mucosa of non-pregnant mice which was negative for CD127 expression (38), highlighting the variability of uterine ILCs since CD127 (IL-7Rα) is also considered to be important for ILC development (6).

In mice, uterine ILC1s are increased throughout gestation compared to the non-pregnant state (37, 39, 40). The production of IFNγ by stimulated total uterine ILCs is increased during gestation (40), which would suggest that these cells have an enhanced capacity for activation in this reproductive tissue. Indeed, uterine ILC1s were shown to contribute to IFNγ production during pregnancy, although not to the same extent as uterine NK cells (38). Consistent with previous findings (42), the uterine ILC1 population was not affected by the knockout of *Nfil3* (38); indeed, ILC1s were increased in these mice (38, 39), indicating that alternative developmental pathways exist for such cells. Since *Nfil3* is crucial for expression of *Eomes* (47), a transcription factor associated with NK cells (48), it was proposed that the uterine ILC1 population observed in *Nfil3*^−/^^−^ mice includes developmentally arrested NK cells (38). However, these residual ILC1s may not be sufficient for mediating uterine adaptation during pregnancy since placental and fetal abnormalities are observed in *Nfil3*^−/^^−^ mice (39).

In humans, ILC1s are found in the non-pregnant endometrium as a subset of Lin-CD56+CD127-CD117-RORγt-cells, which are further distinguished based on expression of NKp44 and CD103 (37). ILC1-like cells, which are CD103+NKp44–, are the most significant source of IFNγ (37). Expression of CD103, which facilitates the communication between lymphocytes and epithelial cells (49), was previously described on tonsillar ILC1s (50), suggesting an epithelial association of such cells in the uterus. However, additional research is needed to determine the role of uterine ILC1s prior to pregnancy and whether such cells are present during gestation.

## Uterine ILC2s

A low frequency of ILC2s has been detected in the human non-pregnant uterine wall (38, 51). In addition, ILC2s and ILC2-like cells have been identified in the non-pregnant murine myometrium (38, 40, 41). The ILC2-like population is the most abundant ILC subset in the pregnant murine myometrium (40). The proportions of murine uterine ILC2s and ILC2-like cells are higher during pregnancy compared to the non-pregnant state, reaching their peak during mid-gestation (38, 40, 51). However, the uterine ILC2-like population (CD45+Lin-Thy1.2+RORγt-NKp46-KLRG1+ cells) identified by Li et al. may have also included other cell types since conventional ILC2 markers such as GATA3 (16) or CRTH2 (52) were not included (40). A recent study utilizing conventional ILC2 markers identified a population of CD127-ILC2s in the non-pregnant human endometrium and in both the non-pregnant and pregnant murine myometrium, confirming the presence of such cells (51). Total uterine ILCs expressing IL-5 and IL-13 were increased during gestation (40), supporting functional roles for uterine ILC2s such as promotion of homeostatic immune cell phenotypes and resolution of inflammatory responses (51).

Uterine ILC2s are almost completely ablated in *Nfil3*^−/−^ mice as opposed to the other subsets (38, 39), confirming that these cells are developmentally reliant on this transcription factor. It is possible that the placental and fetal changes observed in *Nfil3*^−/−^ mice are due to the loss of ILC2-dependent regulatory mechanisms in the myometrium (39); however, since conventional NK cells were also greatly impacted in such mice (38, 39), this finding will require further studies to confirm.

It was recently shown that murine uterine ILC2s express the IL-33 receptor, ST2 (IL-1RL1) (41). A previous report highlighted the importance of IL-33/ST2 signaling for homeostatic immune responses such as those mediated by T helper 2 cells, regulatory T cells, M2-polarized macrophages, and ILC2s, among others (53). In line with these findings, uterine ILC2s were increased in proportion after *in vitro* stimulation with IL-33 (41). ILC2 activity was also increased by *in vitro* IL-33 stimulation as indicated by enhanced release of IL-5 and IL-13 (41). Moreover, an IL-5 reporter mouse (54) was used to verify that *in vivo* administration of IL-33 increased uterine ILC2 proportions and expression of IL-5 (41). Interestingly, the original research describing the IL-5 reporter mouse model demonstrated that the majority of IL-5+ cells in different murine tissues had an ILC2 phenotype, including expression of CD127 and ST2 (54), providing further evidence that IL-33-receptive ILC2s are important for the production of IL-5. Pups born to *ST2*^−/−^ dams had significantly reduced viability (41), suggesting that this pathway may be beneficial for fetal development; however, *IL33*^−/−^ mice do not experience any fertility or pregnancy complications (55). Additionally, IL-33 is important for type 2 mucosal immune responses (55). Together, these observations support pregnancy-specific functions for IL-33-receptive ILC2s in the murine uterus.

Murine uterine ILC2s can also express the estrogen receptor α (41). The proportion of these cells is increased in response to *in vitro* stimulation with 17β-estradiol; however, such a response is not seen in ILC2s from the murine lung (41), providing evidence for specific female sex hormone-driven regulation of uterine ILC2s during pregnancy. Yet, whether female sex hormones specifically target ILC2s, or the observed ILC2 proliferation was a secondary response due to signaling within the uterine tissues, has not been shown (41).

Collectively, these findings provide firm evidence of ILC2s in the non-pregnant uterine tissues from humans and mice, and that such cells are enhanced in number and function during murine gestation. Further studies are required to uncover the specific mechanisms and cellular interactions of uterine ILC2s.

## Uterine ILC3s

ILC3s were first described in the human non-pregnant endometrium as a distinct subset of NK precursor-like cells expressing ILC-associated markers such as CD127 and CD161 (36). Further analysis of these cells revealed expression of the *RORC* and *IL22* genes, indicative of an ILC3 phenotype (36). Later studies confirmed the presence of ILC3s in the human endometrium (37, 38) and indicated that these cells could be divided into two main subsets: NCR– (human NKp44-; mouse NKp46-) and NCR+ (human NKp44+; mouse NKp46+) ILC3s (7), with the NCR– ILC3s being the dominant population in mice and the NCR+ ILC3s in humans (38). During murine pregnancy, uterine ILC3s are elevated compared to non-pregnant mice (38) with the highest proportions occurring in early- and mid-gestation (40). Uterine ILC3s from both pregnant and non-pregnant mice constitutively produce IL-17A and IL-22, which is further upregulated in response to *in vitro* stimulation with IL-1β and IL-23 (38). Yet, production of IL-17A and IL-22 by uterine ILC3s from pregnant mice is not significantly elevated in mid-gestation compared to that of non-pregnant mice (38, 40), suggesting that either an increase in ILC3-specific functionality is not required for successful pregnancy, or that such an increase may only occur in late gestation/prior to parturition. Further studies are required to pursue this concept.

In contrast with ILC2s, the uterine ILC3 population is not affected in non-pregnant *Nfil3*^−/−^ mice (38, 39); however, such cells fail to undergo the pregnancy-specific expansion observed in wildtype mice (39). This lack of ILC3 expansion is associated with fetal growth compromise and defective placentation (39), indicating that uterine ILC3s may be important for the physiological progression of pregnancy, e.g., decidualization (see decidual ILC section for more information).

## Uterine LTi-like cells

Information regarding LTi-like cells in the human and murine uterus is scarce. One potential explanation is that LTi-like cells have been identified as ILC3s due to the shared expression of markers such as RORγt (6). LTi-like cells were reported in the non-pregnant murine uterus in similar proportions to the closely-related ILC3s (38). Moreover, similar to ILC3s, LTi-like cells were not affected in the uterus of non-pregnant *Nfil3*^−/−^ mice (38), suggesting a distinct developmental pathway for these cells. It will be interesting for future studies to uncover the functions of LTi-like cells in the human uterus.

**Figure 1:**
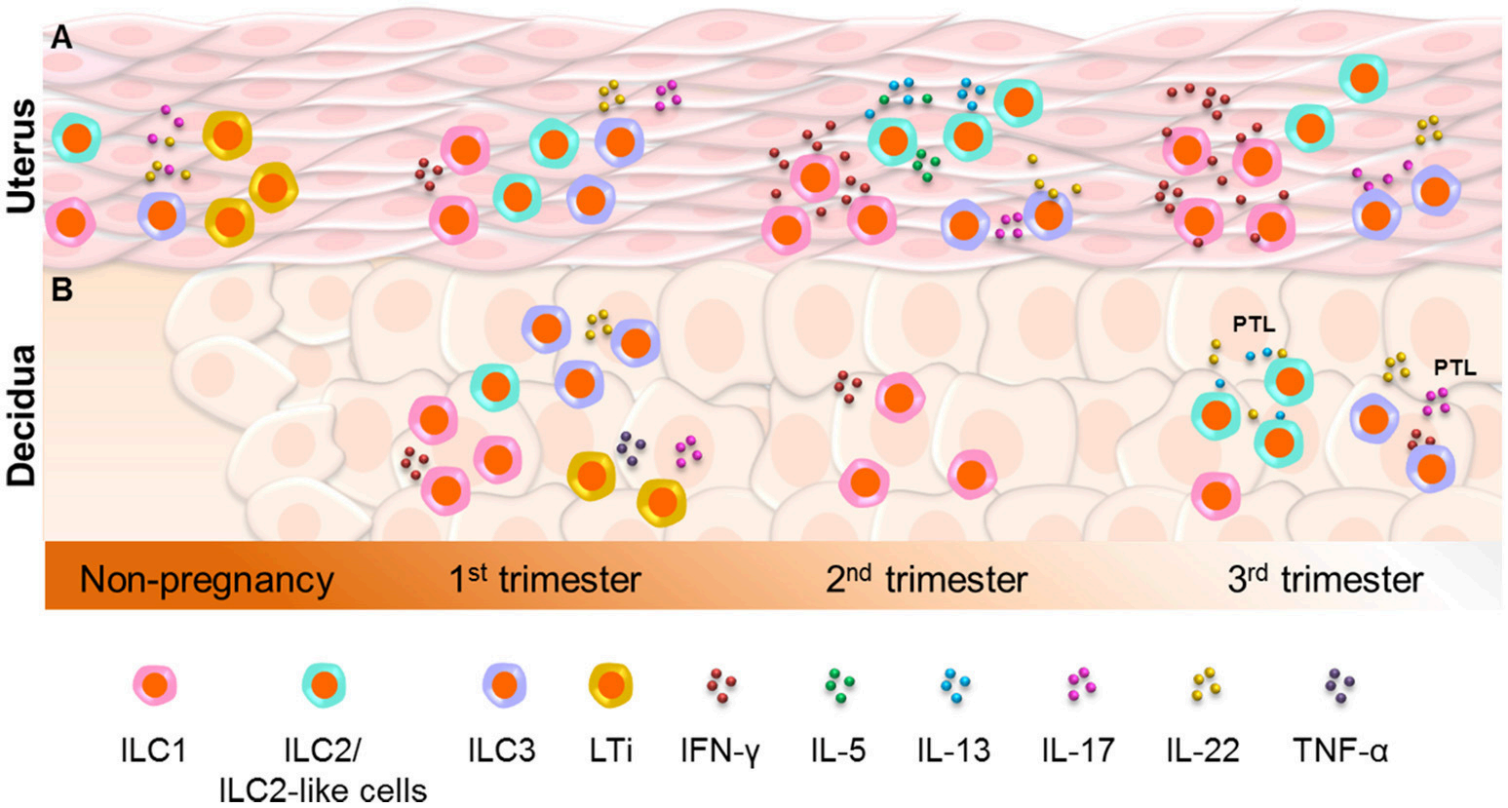
Innate lymphoid cells in the uterus and decidua. **(A)** ILC1s, ILC2s, and ILC3s are present in the murine uterus prior to and during pregnancy, but have only been detected in the non-pregnant endometrium in humans. ILC2s reside in the murine uterus from mid-pregnancy to term, whereas ILC1s increase throughout gestation and ILC3s remain constant. However, lymphoid tissue inducer (LTi) cells have only been detected in the non-pregnant murine uterus. Further studies are needed to confirm the presence and functions of uterine ILCs during human pregnancy. **(B)** In the human decidua, ILC1s, ILC3s, and LTi-like cells are more abundant during early gestation, whereas ILC2s are increased at the end of pregnancy. Decidual ILC1s were also detected during mid-gestation in mice. Interestingly, functional decidual ILC2s and ILC3s are increased in women who underwent spontaneous preterm labor (PTL), indicating the involvement of such cells in this pregnancy complication.

In conclusion (Figure 1A), ILC1s, ILC2s, and ILC3s are present in the murine uterus prior to and during pregnancy, but have only been detected in the non-pregnant endometrium in humans. Specifically, ILC2s reside in the murine uterus from mid-pregnancy to term, ILC1s increase throughout gestation, and ILC3s remain constant. Yet, LTi cells have only been detected in the non-pregnant murine uterus. Further studies are needed to confirm the presence and functions of uterine ILCs during human pregnancy.

## Decidual innate lymphoid cells

Upon implantation, endometrial stromal cells undergo a specialized transformation that includes significant morphological and functional changes to the endometrium, a phenomenon termed as “decidualization” (56, 57). This process facilitates invasion of the fetal trophoblast (56) and leads to formation of the area of contact between the endometrium and the placenta (decidua basalis) or fetal membranes (decidua parietalis). The decidua is therefore an interface in which maternal and fetal cells converge and unique immune interactions take place.

ILCs have been identified in the decidua as early as 9–12 weeks of gestation (58). The origin of decidual ILCs is unclear. Several sources have been proposed for the prototypical ILC1s, NK cells, in the reproductive tissues including derivation from hematopoietic precursors (59), maturation from already-present endometrial NK cells (36), or migration from the periphery (60). Since hematopoietic precursor cells can express the ID2 transcription factor [required for ILC differentiation (61)], this is one plausible explanation for the source of decidual ILCs (59). The origin and developmental timeline of decidual ILCs requires further investigation. Importantly, phenotypic (58) and functional (62) evidence suggests that decidual ILC subsets have unique profiles that are not found in other non-reproductive tissues.

## Decidual ILC1s

ILCs expressing an ILC1-like phenotype distinct from NK cells have been detected in the human decidua during the first trimester (37, 58). Two ILC1 subsets were detected. The first was identified within the CD56+ population (Lin-CD56+CD94-CD127-CD117-) and expressed the ILC1-associated Tbet as well as Eomes (37, 58). Interestingly, this ILC1 subset also expressed CD103 (36, 37), indicating a possible epithelial association (50). The other subset fell within the CD56- population (Lin-CD56-CD127-CD117-Tbet+Eomes-) and was therefore more distinguishable from decidual NK cells (36). In line with defined ILC1 phenotypes, both described subsets expressed IFNγ (37, 58). In mice, ILC1s were also described in the decidua during mid-gestation, where they produced IFNγ (38).

At the end of pregnancy, ILC1s are the rarest ILC subset in the human decidua and were not altered with the presence of spontaneous labor, suggesting that such cells may have only a minor role in late gestation that may be shared by other decidual ILC subsets due to the unique cytokine profile observed in such cells (62).

## Decidual ILC2s

The first-trimester human decidua has been reported to contain a small proportion of ILC2s (38, 51, 58). The expression of the ILC2 marker CRTH2 (52) was only minimally detected on decidual ILCs (38, 58); however, CD161 (52) was expressed by two of the potential ILC subsets described, indicating possible plasticity or shared expression of ILC2 markers by other subsets (58). ILC2s were found in the murine uterus, but not the decidua, in mid-gestation (38, 51) and at term (51). In contrast, ILC2s were the most abundant decidual ILC subset in the third trimester (62) where they may play a role in maintaining the homeostatic environment at the maternal-fetal interface. ILC2s are considered to have a homeostatic phenotype (19, 20), rendering them unnecessary in early pregnancy when the inflammatory mechanisms of implantation and tissue remodeling occur within the endometrium. Interestingly, ILC2s were increased in the decidua basalis of women with spontaneous preterm labor compared to those who delivered preterm without labor (62), suggesting that this ILC subset may participate in the chronic inflammatory process that occurs during pathological pregnancy. Moreover, ILC2s from the third-trimester human decidua seemed to share the expression of cytokines such as IL-13 and IL-22 with ILC3s, suggesting that decidual ILC subsets may have shared functionality toward the end of pregnancy (62).

## Decidual ILC3s

Among the described ILC subsets in the human decidua, ILC3s have been the most extensively studied (37, 38, 58, 62, 63). During the first trimester, a subset of ILCs expressing the traditional ILC3-associated transcription factor RORγt (26) is found in the human decidua (37, 38, 58). Decidual ILC3s expressed GM-CSF (63), IL-22 (58), and IL-8 (58). Notably, GM-CSF and IL-8 released by decidual ILC3s were shown to promote neutrophil migration and survival in the first-trimester decidua (63). This finding is consistent with a previous study that showed the participation of neutrophils in spiral artery remodeling during pregnancy (64), and adds a new layer of complexity to the role of decidual ILC3s in the successful establishment of pregnancy (63). Interestingly, the murine decidua did not contain ILC3s during mid-gestation (38).

During the third trimester, human decidual ILC3s express a unique cytokine profile that includes IFNγ, IL-13, IL-17A, and IL-22 (62). Previous findings have suggested that some degree of plasticity exists between ILC subsets (28, 29, 65), which would explain the expression of the ILC1-associated cytokine IFNγ by decidual ILC3s. This may also explain the low proportions of decidual ILC1s in late gestation (62), since their presence may be redundant.

Interestingly, increased proportions of ILC3s in the decidua parietalis are found in women who undergo spontaneous preterm labor (62), suggesting that a local dysregulation of such cells may occur in these patients. Whether decidual ILC3s directly participate in the inflammation associated with spontaneous preterm labor or are increased as a consequence of such a process remains to be determined.

## Decidual LTi-like cells

Human LTi cells have important functions in the formation of fetal SLO (66), a process described in more detail below; however, their role at the maternal-fetal interface is less understood. A population of LTi-like cells has been described in the human first-trimester decidua where they express IL-17A and TNFα (58). These decidual LTi-like cells are closely related to ILC3s as evidenced by the shared expression of RORγt and production of IL-17A (58). Moreover, both decidual LTi-like cells and ILC3s display lymphoid tissue inducer-like functions (22–25, 67–69) through the upregulation of ICAM-1 and VCAM-1 on decidual stromal cells (58), further indicating a degree of redundancy between these two cell types (27, 70). However, the developmental pathways of LTi cells and ILC3s are different and, unlike other ILC subsets, no LTi plasticity has been reported (27). It is possible that the initiation of a lymphoid tissue induction-like process in the decidua is necessary for recruitment of other immune cells and pregnancy maintenance. Therefore, it is important for future studies to investigate the purpose of LTi-like activity at the maternal-fetal interface.

In conclusion (Figure 1B), the human decidua contains ILC1s, ILC3s, and LTi-like cells, which are more abundant during early gestation. In contrast, decidual ILC2s are increased at the end of pregnancy. Decidual ILC1s were also detected during mid-gestation in mice. Functional decidual ILC2s and ILC3s are increased in women who underwent spontaneous preterm labor, indicating the involvement of such cells in this pregnancy complication.

## Fetal innate lymphoid cells

Fetal ILCs are reported to exist in the human liver, SLO, intestine, and lung, which are described in detail below. The fetal liver is a center of hematopoiesis (71, 72), and it has been shown that ILC progenitors (ILCP) originate from this compartment (65, 73–76). Indeed, ILCPs can be detected in the cord blood as well, indicating that such cells may migrate to other sites of organogenesis (76). It has been proposed that the differentiation of ILCPs to mature ILC subsets primarily takes place after such cells have migrated to their sites of residence (76). In the fetus, the presence of specialized ILCs (i.e., LTi cells) is important for the successful formation of SLO such as the spleen, mesenteric lymph nodes (mLN), and Peyer's patches (23, 26, 77, 78). At non-lymphoid sites such as the intestine and lung, mature ILC subsets may participate in mucosal immunity after birth by regulating inflammation during colonization with commensal bacteria (79, 80).

In mice, development of the fetal lymphatic system is described as early as gestational day 10.5 (81). The murine fetal lymph nodes follow a staggered developmental timeline beginning with the mLN at gestational day 10.5, closely followed by the sacral and cervical lymph nodes and ending with the complete formation of Peyer's patches in the intestine [for more information about fetal lymphogenesis, please see (82, 83)]. The mesenteric and peripheral lymph nodes are present in the fetus by gestational day 16.5 (68). ILCPs are detected in the murine fetal liver at day 12.5 (84–88), and in both the fetal liver and intestine at day 13.5–14.5 (74, 89). Information regarding the presence of ILCPs in the fetal tissues in early pregnancy is lacking; therefore, further studies are required to determine the complete timeline for the generation of ILCPs and mature ILC subsets during fetal development.

## ILCs in the fetal liver

A subset of ILCPs was described in the human fetal liver during the second trimester (76). It was shown that these ILCPs were generated from the CD34+ hematopoietic stem cells (76) also found in this compartment (71, 72, 76). These ILCPs primarily express RORγt and, after *in vitro* expansion, mainly produce IL-17A, indicating an ILC3 phenotype (76). However, subsets of fetal liver ILCPs also produce IFNγ or IL-13, suggesting that such cells have differentiation potential for ILC1s and ILC2s as well (76). The murine fetal liver also contains an ILCP subset with potential for differentiation into ILC1s, ILC2s, or ILC3s (75). Ablation of *Zbtb16*, which encodes the ILCP-associated transcription factor PLZF [reviewed in (88)], affected fetal ILC1s and ILC2s but not ILC3s or NK cells (75), supporting the existence of alternative progenitors or developmental pathways for these ILC subsets.

The human fetal liver also contains mature ILC populations during the first and second trimester (90). ILC1s, ILC2s, and both NCR+ and NCR– ILC3s are detected (90). Prior to 15 weeks of gestation, only NCR– ILC3s can be distinguished, whereas the remaining subsets appear later (90). A population of fetal liver ILC3s express neuropilin-1 (NRP-1) (90, 91), suggesting an LTi phenotype (92). Together, these findings indicate that the fetal liver is the primary site of ILC progenitors. Mature ILC subsets also exist within the fetal liver, yet their role is currently unknown.

## ILCs in the fetal lymphoid tissues

Murine experiments have shown that the interaction between LTi cells and mesenchymal stromal cells is fundamental for the formation of SLO (93). It has been observed that during murine embryogenesis a subset of stromal cells interacts with LTi cells at the site of LN formation (25, 26, 68). LTi cells express ligands such as lymphotoxins α and β (LTA and LTB) that activate specific stromal cells (25, 94–96). Such activated stromal cells will upregulate expression of the adhesion molecules ICAM-1 and VCAM-1 (25, 69, 78, 97) and begin the process of forming SLO (26).

A subset of Lin-CD127+ ILCs was originally described in human fetal mesenteric tissue (97). It was shown that these ILCs were localized at the same locations at which the mLN developed (91, 97), indicating that lymph node-specific ILCs are present in the fetal mesentery even prior to the complete formation of the mLN. Moreover, stromal organizer cells form a niche for LTi cells in the human fetal spleen and LN between 8 and 15 weeks of gestation (78), providing further evidence of an important role for fetal LTi cells in tissue neogenesis. The mLN from first- and second-trimester human fetuses have been shown to contain an ILC subset that expressed RORγt and had increased gene expression of *IL17A* and *IL22* (98), and a similar subset was described in the fetal spleen that also expressed NRP-1 (91, 92). *NRP1*^−/−^ knockout mice have severely affected yolk sac and embryonic development (99), suggesting that the expression of this receptor is required for organogenesis. Additionally, RORγt+ ILCs are found in specific physiological locations in the human fetal LN and spleen in the second trimester, where they are co-localized with specialized stromal cells (78). This interaction leads to induced expression of ICAM-1 and VCAM-1 on the stromal cells (78, 97), indicating that these ILCs have LTi functions. LTi cells in the human fetal LN express *IL17A* and *IL22* and participate in LN formation (97).

## ILCs in the fetal intestine

LTi cells cluster at the site where Peyer's patches are formed in the developing murine fetal intestine (22, 89). The development of intestinal lymphoid tissues such as the Peyer's patches is imperative for regulation of mucosal immunity in the intestine (100, 101), and fetal LTi cells have been shown to be crucial to this process (23, 26). Importantly, a subset of transitional ILCPs exists in the fetal intestine that can further differentiate into other ILC subsets, indicating that some of these ILCs are not terminally differentiated and can provide other functions even after SLO formation is complete (89).

Recently, the presence of mature ILC subsets in the fetal intestine was confirmed using mass cytometry (102). All known ILC subsets were detected together with several novel intermediates that included a subset with potential to differentiate into ILC3s or NK cells (102). These findings confirmed previous studies that indicated the presence of ILC2s (52) and ILC3s (52, 98, 103) in the second-trimester human fetal intestine. ILC2s in the fetal intestine produce IL-13 (52), whereas ILC3s and LTi-like cells produce IL-17A and IL-22 (98). ILC3s are increased in the fetal intestine during the second trimester compared to the first (98, 103). Importantly, fetal CD103+ ILC3s can be found in the amniotic fluid during the first and second trimesters (see amniotic cavity section for more information) (103), suggesting that these cells can migrate from the fetal tissues into the amniotic cavity.

ILCs are increased in the intestinal tissues from neonates with gastroschisis compared to those from healthy controls (104). This finding was corroborated using a murine model with gastroschisis-like symptoms showing that ILC2s and ILC3s are increased in the intestines of affected mice compared to littermate controls (104). Neutralization of IL-5 [a primary ILC2 cytokine (13)] during late gestation results in a dramatic decrease in eosinophil and ILC2 infiltration in the fetal intestine (104), implicating ILC2s in the chronic inflammatory process that accompanies this condition.

Collectively, these data confirm the requirement for LTi cells during the formation of fetal SLO, and indicate that mature ILC1s, ILC2s, and ILC3s are found in the intestinal mucosa where they may participate in inflammatory processes; however, their specific role during fetal life is unclear.

## ILCs in the fetal lung

A single report established the presence of ILC2s (identified by CRTH2 and CD161 expression) in the human fetal lung mucosa during the second trimester (52). In the neonatal and adult lungs, the primary function of ILC2s is to protect against threats such as helminth infection (12, 13, 105). ILC2s are also implicated in asthma and its complications as well as allergy (18, 106, 107). A subset of CD103+ ILC3s is also detected in the human fetal lung, which is increased in the second trimester compared to the first (103). Recently, a population of ILC2s was described in the murine fetal lung just prior to birth (gestational day 19), which rapidly expanded during the first 2 weeks of life (108). It was suggested that these homeostatic cells may help prevent hyper-inflammation resulting from exposure of the newborn lungs to airborne particles (108). Future studies may further reveal the specific role of ILC2s and ILC3s in this fetal compartment.

**Figure 2:**
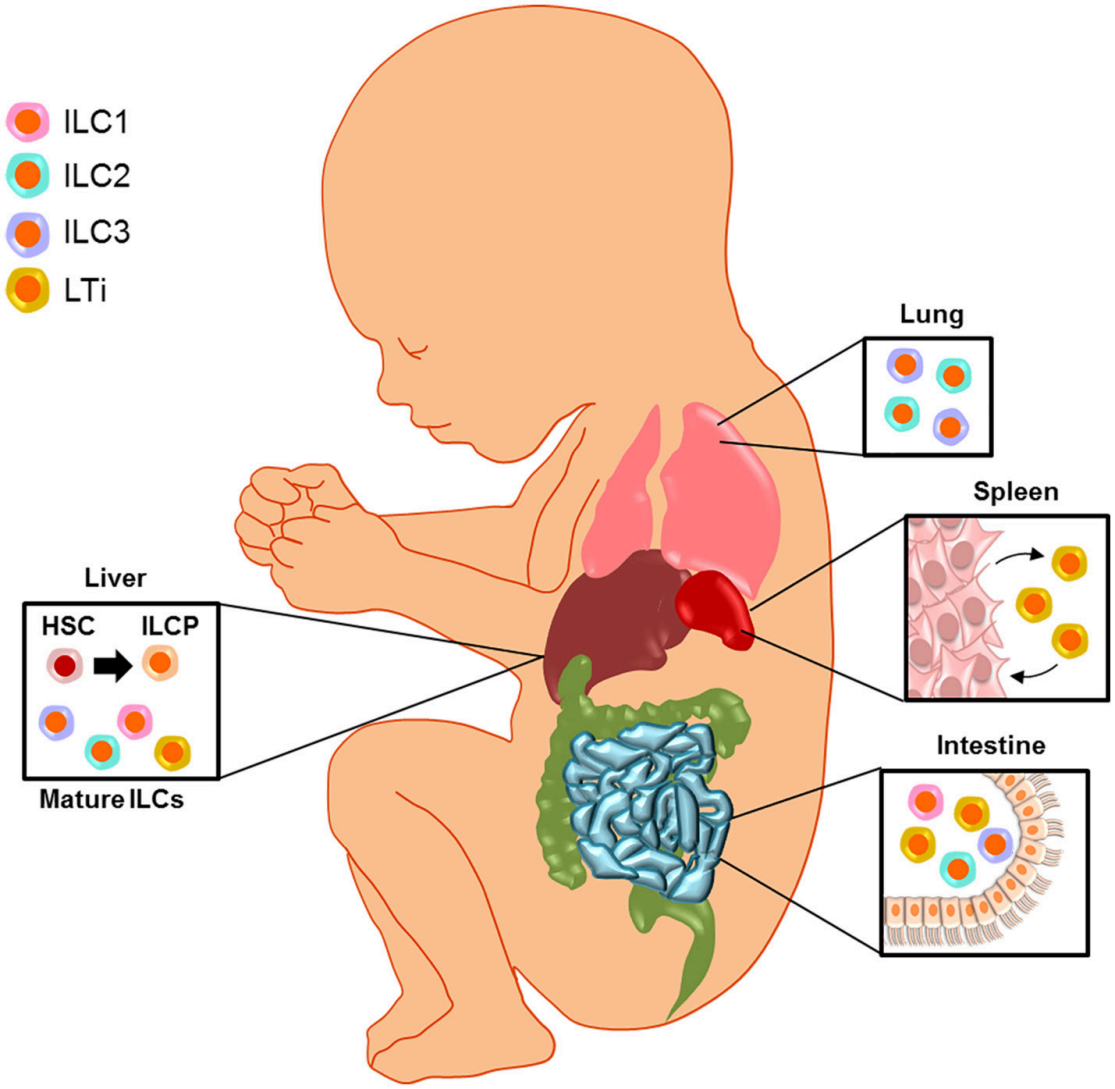
Fetal innate lymphoid cells. Fetal ILCs exist in the liver, secondary lymphoid organs (SLO), intestine, lung, and amniotic cavity. The fetal liver is thought to be the source of ILC progenitors (ILCP) since the differentiation of these cells from hematopoietic stem cells (HSC) occurs at this site, and mature ILC subsets can be found in this compartment as well. The interaction between lymphoid tissue inducer (LTi) cells and specialized stromal cells is important during the formation of SLO. Mature ILCs are found at the mucosal surfaces of the lung and intestine, from where they can extravasate into the amniotic cavity. These findings support a role for ILCs as central regulators in fetal development and immunity.

In conclusion (Figure 2), fetal ILCs exist in the liver, SLO, intestine, lung, and amniotic cavity. The fetal liver is thought to be the source of ILC progenitors since the differentiation of these cells from hematopoietic stem cells occurs at this site, and mature ILC subsets can be found in this compartment as well. The interaction between LTi cells and specialized stromal cells is important during the formation of SLO. Mature ILCs are found at the mucosal surfaces of the lung and intestine, from where they can extravasate into the amniotic cavity. These findings support a role for ILCs as central regulators in fetal development and immunity.

## Innate lymphoid cells in the amniotic cavity

The amniotic cavity serves as the fetal habitat, which is surrounded by the protective liquid termed amniotic fluid (109). Besides providing mechanical cushioning, the amniotic fluid contains nutrients as well as other factors required for fetal growth and represents an immunological barrier against invading pathogens (109, 110). In clinical medicine, the amniotic fluid is used to assess fetal well-being (111–114), lung maturity (115–117), karyotype (118, 119), and intra-amniotic inflammation associated with bacteria [intra-amniotic infection (120–132)] or danger signals [sterile intra-amniotic inflammation (133–138)]. In the context of intra-amniotic inflammation, the most abundant leukocytes in the amniotic fluid are neutrophils (139, 140), which can be of fetal and/or maternal origin (141, 142). These innate immune cells actively participate in the mechanisms of host defense against microbial invasion of the amniotic cavity by releasing cytokines (140) and anti-microbial molecules (143–145), performing phagocytosis (146), and forming neutrophil extracellular traps or NETs (147, 148). Therefore, it was thought that, in the absence of intra-amniotic inflammation, the cellular component of the amniotic fluid was of limited research value. Recent studies have shown that, indeed, the opposite is true (103, 149). The amniotic fluid contains both innate (monocyte/macrophages, neutrophils, NK cells, and ILCs), and adaptive (T cells and B cells) immune cell populations, each of which fluctuates independently throughout gestation (149).

**Figure 3:**
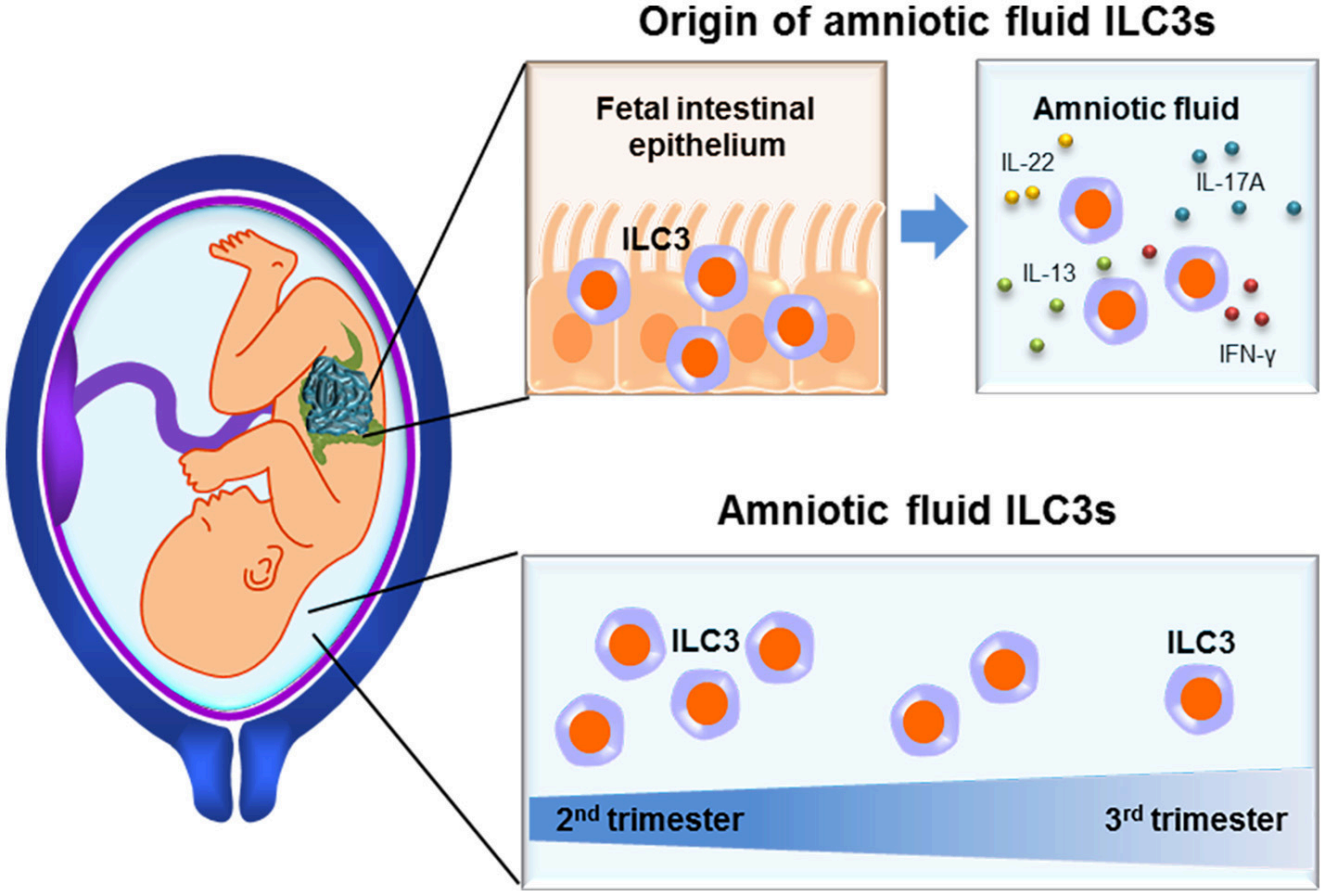
Innate lymphoid cells in the amniotic cavity. Functional ILC3s are found in the mucosal epithelium of fetal tissues such as the intestines, from where they may extravasate into the amniotic cavity. Amniotic fluid ILCs reach their highest proportions during the second trimester, yet are still present at the end of gestation.

Amniotic fluid ILCs are abundant during the second trimester (103) and their numbers decay as gestation progresses (149) (Figure 3). In this compartment, ILCs express high levels of RORγt (103, 149), a hallmark of ILC3s (6, 26). Amniotic fluid ILC3s also express CD127, CD117, CD161, and CD56 but not NK cell-markers such as Eomes, T-bet, CD94/NKG2A, and CD16 (103). Such ILCs are functional since they produce high levels of IL-17A and TNFα upon PMA/ionomycin stimulation (103). The fetal origin of amniotic fluid ILC3s was demonstrated by the expression of HLA class I molecules, which were not expressed on maternal peripheral blood mononuclear cells (103). Interestingly, amniotic fluid ILC3s seem to originate in the fetal lungs and intestine since a similar ILC subpopulation was identified in these organs (103). Amniotic fluid ILC3s expressed CD103, indicating an epithelial association (49, 50) that was confirmed by detection of these cells in the fetal intestinal epithelium (103). Moreover, CD103+ ILC3s were not detected in the amnion or chorion (chorioamniotic membranes), eliminating those tissues as a source of such cells in the amniotic fluid (103). Together with the observation that immune cells in the amniotic fluid during preterm gestation can be predominantly of fetal origin (142), evidence points to the fetus as a likely source of CD103+ ILC3s (Figure 3). It was proposed that these cells participate in regulating intra-amniotic infection (103); yet, their numbers remain constant between patients with and without this clinical condition (149). This finding does not discard the possibility that amniotic fluid ILC3s can acquire a regulatory phenotype, which can then participate in controlling the inflammatory response induced by microbes or danger signals in the amniotic cavity.

Together, these studies demonstrate the presence of functional ILC3s in the amniotic cavity, which are likely derived from the fetal tissues. Such cells reach their highest proportions in the second trimester yet are still present at the end of gestation. Moreover, the detection of ILC3s in the amniotic cavity of patients with intra-amniotic inflammation suggests the participation of these cells in such a clinical condition.

Molecular studies have suggested that there is a placental microbiome (150–161). Nonetheless, recent publications have not confirmed that the placenta harbors a unique microbiome [(162); Theis et al., Am J Obstet Gynecol; in press], which supports the ongoing controversy [(162–166); Theis et al., Am J Obstet Gynecol; in press]. The absence of a placental microbiota, however, does not exclude the possibility that the fetus is exposed to microbial products from the mother. This concept is supported by another recent study showing that transient microbial colonization of the maternal gut during pregnancy induces short- and long-term innate immune changes in the offspring (167). Of interest to the ILC field, neonates born to mothers transiently microbial-colonized displayed an increased number of ILC3s in their mucosal tissues (167). The proposed mechanism for fetal exposure to maternal gut microbiota was mediated by transmission of microbial-derived metabolites via antibodies (167). Such education of the neonatal immune system was enhanced by breastfeeding (167). Therefore, microbial-derived metabolites, rather than viable bacteria, may be required for fetal and neonatal development of the ILC system.

## Conclusion

The discovery of ILCs in the reproductive and fetal tissues has led to new knowledge of the immune cellular processes required for successful pregnancy and fetal development. At the same time, new questions have arisen as to the functions and interactions of ILCs in the maternal and fetal compartments. The studies reviewed herein have provided evidence that ILCs fill an important role during pregnancy, especially in mucosal defenses and fetal development, yet also share certain functions with other innate and adaptive immune cell subsets. In the mother, uterine ILCs may participate in mucosal immunity and help facilitate tissue remodeling and homeostasis during and after implantation, while decidual ILCs take part in the immune interactions required for pregnancy maintenance and maternal-fetal tolerance. Meanwhile, fetal ILCs mediate the formation of lymphoid tissues during organogenesis and reside at key mucosal surfaces, such as the intestine and lung, in preparation for fetal exposure to both commensal and pathogenic microbes. Importantly, such fetal ILCs may migrate to the amniotic fluid during intra-amniotic infection/inflammation to further participate in host defense. Collectively, the presented findings paint a complex picture of the ILC network during pregnancy, and future studies will be required in order to reveal the complete story of these unique immune cells.

## Author contributions

DM, KM, VG-F, RR, and NG-L participated in the conception, interpretation, and writing of the manuscript.

### Conflict of interest statement

The authors declare that the research was conducted in the absence of any commercial or financial relationships that could be construed as a potential conflict of interest.
